# The FIB-4 Index Is Independently Associated with QTc Interval in Patients with Ankylosing Spondylitis

**DOI:** 10.3390/jcm15020595

**Published:** 2026-01-12

**Authors:** Elif Ergül, Hüseyin Durak, Mustafa Çetin, Hakan Duman, Nadir Emlek, Ahmet Seyda Yılmaz, Ali Gökhan Özyıldız, Gökhan Barutçu, Osman Cüre

**Affiliations:** 1 Department of Cardiology, Faculty of Medicine, Recep Tayyip Erdoğan University, 53020 Rize, Türkiye; 2 Department of Cardiology, Elmalı State Hospital, 07700 Antalya, Türkiye; 3 Department of Rheumatology, Faculty of Medicine, Recep Tayyip Erdoğan University, 53020 Rize, Türkiye

**Keywords:** QTc, FIB-4 index, ankylosing spondylitis

## Abstract

**Objective:** Prolongation of the QTc interval (QTc) is a known risk factor for ventricular arrhythmias and sudden cardiac death (SCD). Although ankylosing spondylitis (AS) is associated with systemic inflammation and metabolic alterations, data on the relationship between noninvasive fibrosis markers and QTc are limited. This study aimed to investigate the association between the FIB-4 index and QTc in patients with AS. **Methods:** A total of 82 consecutive patients with AS were enrolled in the study. Demographic characteristics, comorbidities, laboratory parameters, and medication use were also recorded. The FIB-4 index was calculated for each patient in the study. Surface 12-lead electrocardiograms were obtained, and the QTc was measured. Correlation analyses and multivariable linear regression models were used to identify the independent predictors of QTc. **Results:** The mean age of the study population was 42.4 ± 11.7 years, and 57.3% of the patients were men. Correlation analysis revealed significant associations between QTc and age, sex, the FIB-4 index, body mass index (BMI), hypertension, hyperlipidemia, and cardiovascular medication use, whereas hemoglobin and estimated glomerular filtration rate (eGFR) were negatively correlated with QTc. In the multivariable analysis, only sex (β = −0.306, *p* = 0.001) and the FIB-4 index (β = 0.379, *p* < 0.001) remained independently associated with QTc. **Conclusion:** Our findings demonstrate that the FIB-4 index is independently associated with the QTc in patients with AS. These results suggest that noninvasive fibrosis markers may provide additional insights into cardiovascular risk stratification in this population.

## 1. Introduction

Ankylosing spondylitis (AS) is a systemic chronic inflammatory disease of the axial skeleton that predominantly affects young men and involves both skeletal and extraskeletal structures [1,2]. Cardiac involvement is a well-recognized complication of AS, with aortic root disease being the most common manifestation. However, recent studies have shown that systemic inflammation in patients with AS may also affect various cardiac components, including the heart valves, conduction system, myocardium, and vasculature [3].

The QT interval on electrocardiography (ECG) represents the time from the onset of ventricular depolarization to the completion of repolarization of the ventricles. The American Heart Association (AHA) recommends correcting the QT interval (QTc) for heart rate, most commonly using Bazett’s formula [4]. Because it is simple to measure and interpret, the QT interval is frequently used to assess cardiac electrical and autonomic function in studies on rheumatic diseases [5,6,7].

Non-alcoholic fatty liver disease (NAFLD) is the most common cause of chronic liver disease worldwide. It encompasses a wide spectrum of hepatic abnormalities, with advanced fibrosis known to confer a worse prognosis [8,9]. Among the noninvasive fibrosis scores used in clinical practice, the Fibrosis-4 (FIB-4) index is one of the most widely used tools [10].

In the present study, we aimed to evaluate the relationship between the FIB-4 index, a noninvasive and easily calculated score derived from clinical and laboratory variables, and QTc in patients with AS.

## 2. Methods

This was an observational and cross-sectional study. A total of 82 consecutive patients with a prior diagnosis of AS established at the rheumatology clinic were included.

Patients were excluded if they had concomitant medical or surgical conditions known to cause QT interval prolongation; cardiac diseases associated with prolonged QT; bundle branch block or nonspecific intraventricular conduction delay with a QRS duration ≥120 ms; a history of cardiac arrest; chronic viral hepatitis; previous or current biliary obstruction; chronic alcohol consumption; severe renal impairment (eGFR < 30 mL/min/1.73 m^2^); moderate-to-severe valvular heart disease; severe hematologic disorders; active inflammatory disease; malignancy; previous liver transplantation; or marked frailty in advanced age.

After a 12 h overnight fast, venous blood samples were collected to obtain the biochemical measurements required for calculating the FIB-4 index and other laboratory parameters. Demographic and clinical data, including age, sex, and medication use, were obtained through a structured patient interview and a detailed standardized form, in addition to review of hospital records. Diabetes mellitus (DM), hypertension (HT) and hyperlipidemia (HL) were defined according to the current guidelines [11,12,13]. The FIB-4 score was calculated based on laboratory tests using the following formula:

FIB-4 = (Age [years] × AST [U/L])/(Platelet count [×10^9^/L] × √ALT [U/L])

Standard 12-lead ECGs (Cardiovit AT-10 Plus ECG system, SCHILLER AG, Baar, Switzerland; filter 150 Hz, paper speed 25 mm/s, amplitude 10 mm/mV) were recorded for all patients in the supine position after at least 10 min of rest. Standard intervals and amplitudes were measured by an experienced cardiologist who was blinded to the patients’ clinical information. The QT interval was measured in all 12 leads, and the longest value was used for the analysis. The QT intervals were corrected for heart rate using Bazett’s formula (QTc = QT/√RR) to obtain the QTc.

The diagnosis of ankylosing spondylitis was made by a rheumatologist based on the Assessment of Spondyloarthritis International Society (ASAS) classification criteria [14]. Patients fulfilled the ASAS criteria for axial spondyloarthritis, including clinical features, imaging findings (sacroiliitis on MRI and/or radiography), and laboratory parameters where applicable. Conventional radiography (X-ray) and magnetic resonance imaging (MRI) have been used in routine clinical practice to evaluate the sacroiliac joints. However, only patients with sacroiliitis findings detected on MRI were included in this study. Conventional radiography findings were not included in the evaluation criteria of the study due to their limited sensitivity in demonstrating early inflammatory changes. Non-radiographic axial spondyloarthritis (nr-axSpA) patients were included if sacroiliitis was detected on MRI in accordance with ASAS criteria; X-ray findings were not used for inclusion.

### 2.1. Ethics Approval and Informed Consent

The study was conducted in accordance with the principles of the Declaration of Helsinki and approved by the Institutional Ethics Committee of Recep Tayyip Erdoğan University (approval date: 7 November 2024 approval number: E-34212324-205.07.11-7). Written informed consent was obtained from all participants prior to enrollment.

### 2.2. Statistical Analysis

All statistical analyses were performed using the SPSS software (version 23.0; SPSS Inc., Chicago, IL, USA). The distribution of variables was assessed using visual methods (histograms and probability plots) and analytical tests (Kolmogorov–Smirnov and Shapiro–Wilk tests). Levene’s test was used to evaluate the homogeneity of variances. Continuous variables are presented as mean ± standard deviation, and categorical variables are expressed as percentages. Non-normally distributed variables are reported as median and interquartile range. Categorical groups were compared using the chi-square test or Fisher’s exact test when the chi-square assumptions were not met due to low expected cell counts. Normally distributed continuous variables were compared using one-way ANOVA, whereas the Mann–Whitney U test was applied for non-normally distributed variables.

Variables showing significant differences between groups (*p* < 0.05) were first entered into a univariate analysis. Parameters that remained statistically significant were included in a backward multivariable logistic regression model (* indicates variables included in the model).

### 2.3. Figure Generation

All figures were produced in PNG format at 300 DPI, using a color-blind-friendly palette and Arial font. Graphics were generated in Python(3.14.2 version) using the Matplotlib(version 3.10.7) and Seaborn(version 0.13.2) libraries. All the plotting steps were documented to ensure reproducibility.

## 3. Results

A total of 82 patients were included in this analysis. The mean age of the participants was 42.4 ± 11.7 years, and 57.3% (*n* = 47) were men. Regarding comorbid conditions, DM was present in 15.9% of patients, HT in 22%, HL in 52.4%, and coronary artery disease in 4.9%. Current smoking was reported by 38.3% of participants. The mean BASDAI score was 4.1 ± 2.3, the mean FIB-4 index was 0.75 ± 0.32, the mean eGFR was 105.6 ± 15.9 mL/min/1.73 m^2^, and the mean serum creatinine level was 0.78 ± 0.17 mg/dL (Table 1).

**Figure 1:**
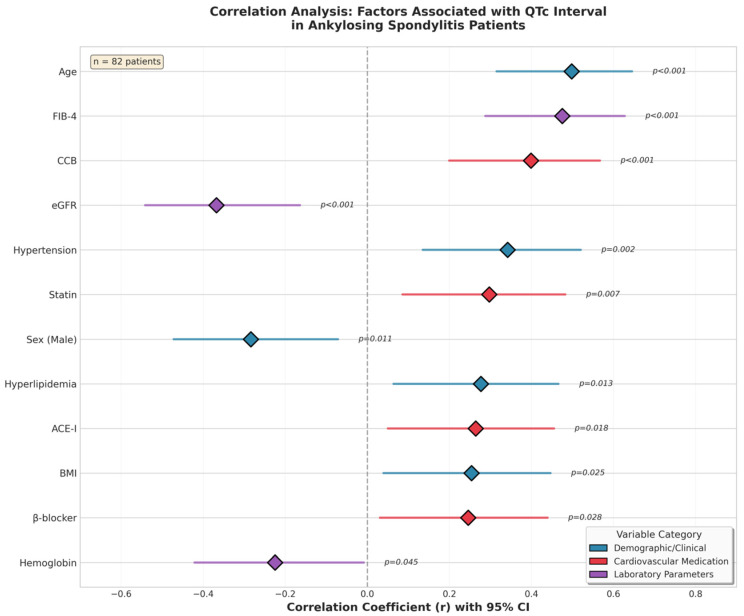
Correlation analysis of factors associated with QTc interval in patients with ankylosing spondylitis.

**Figure 2:**
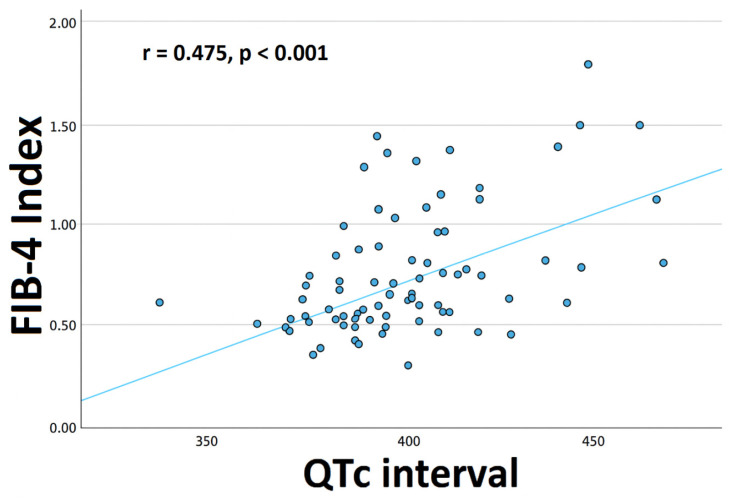
Scatter plot of the association between the FIB-4 index and the QTc interval in patients with ankylosing spondylitis.

Correlation analysis revealed significant positive associations between QTc and age (r = 0.498, *p* < 0.001), sex (r = −0.284, *p* = 0.011), FIB-4 index (r = 0.475, *p* < 0.001), body mass index (BMI) (r = 0.254, *p* = 0.025), HT (r = 0.342, *p* = 0.002), HL (r = 0.277, *p* = 0.013), β-blocker use (r = 0.246, *p* = 0.028), calcium channel blocker (CCB) use (r = 0.399, *p* < 0.001), angiotensin converting enzyme inhibitor (ACE-i) therapy (r = 0.264, *p* = 0.018), and statin use (r = 0.297, *p* = 0.007). Hemoglobin levels (r = −0.225, *p* = 0.045) and estimated glomerular filtration rate (eGFR) (r = −0.368, *p* < 0.001) were significantly negatively correlated with QTc (Table 2). The correlation analysis of factors associated with the QTc interval is shown in Figure 1. A scatter plot of the association between the FIB-4 index and the QTc in patients with AS is shown in Figure 2.

**Figure 3:**
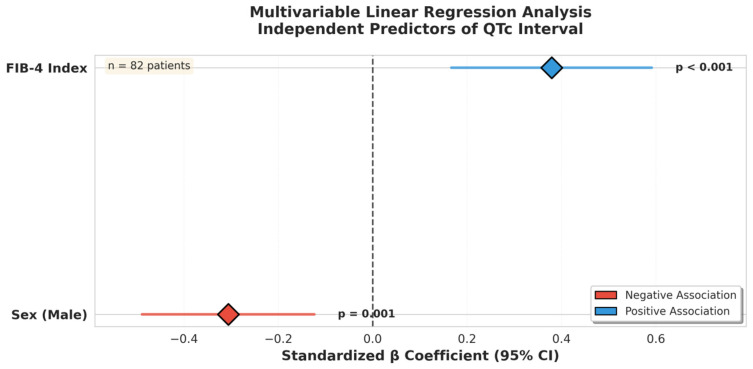
Multivariable linear regression model showing independent predictors of QTc interval.

In the multivariable linear regression analysis, sex (β = −0.306, *p* = 0.001) and the FIB-4 index (β = 0.379, *p* < 0.001) were identified as independent predictors of QTc. These findings indicate that the FIB-4 index is independently and significantly associated with QTc prolongation (Table 3). Figure 3 shows the multivariable linear regression model of the independent predictors of the QTc.

This figure displays the correlation coefficients (r) and 95% confidence intervals for demographic variables, cardiovascular medications, and laboratory parameters associated with QTc interval in ankylosing spondylitis. Positive and negative associations are shown along a horizontal axis, with statistically significant variables indicated by corresponding *p*-values. Color coding denotes variable categories (clinical/demographic, cardiovascular medication, laboratory parameters). Analysis was based on 82 patients.

This scatter plot illustrates the positive correlation between the FIB-4 index and the QTc interval in 82 patients with ankylosing spondylitis. Each data point represents an individual patient, and the fitted regression line demonstrates that higher FIB-4 values are associated with longer QTc intervals.

This figure presents the standardized β coefficients and 95% confidence intervals from the multivariable linear regression analysis. Among all variables entered into the model, only the FIB-4 index showed a significant positive association with the QTc interval, whereas male sex demonstrated a significant negative association. Analysis was performed on 82 patients.

## 4. Discussion

In this study, we found that the FIB-4 index was independently associated with QTc measured using a 12-lead surface ECG in patients with AS.

AS is a chronic rheumatic disease characterized by recurrent inflammation that primarily affects the axial skeleton and related joints. Cardiac abnormalities are well-recognized complications of AS. The most commonly described abnormalities include aortic and mitral regurgitation, atrioventricular and intraventricular conduction disturbances, pericarditis, cardiomyopathy, sinus node dysfunction, and atrial and ventricular arrhythmias [15,16,17,18,19,20,21].

Chronic inflammation is the pathophysiological link between autoimmune diseases and autonomic dysfunction, including sympathetic overactivation and reduced parasympathetic activity [22]. The QT interval is influenced by both sympathetic and parasympathetic branches of the autonomic nervous system [23,24]. Conduction disturbances, an increased frequency of ventricular arrhythmias, and possible impairment of cardiac autonomic function have been reported in patients with AS [17,20]. Taken together, these findings suggest that chronic inflammation in patients with AS may contribute to QT interval prolongation. Yıldırır et al. examined 88 cardiologically asymptomatic AS patients (mean age 33 ± 11 years; mean disease duration 5.6 ± 6 years) and found that ventricular extrasystoles and increased QT dispersion were more frequent in AS patients than in healthy controls [21].

QTc prolongation may lead to syncope, ventricular fibrillation, cardiac arrest, and sudden cardiac death (SCD) through the development of Torsades de Pointes, a form of polymorphic ventricular tachycardia [4]. Studies in patients with rheumatoid arthritis (RA) have demonstrated that systemic inflammation may contribute to QTc prolongation by accelerating the development of structural heart diseases [25,26]. Accelerated atherosclerosis and inflammation-induced myocardial injury and remodeling have been proposed as potential mechanisms through which systemic inflammation may directly promote structural cardiac pathologies [27,28,29].

Several studies have evaluated the direct effects of inflammatory cytokines on the QT interval [30]. The association between systemic inflammation and QTc prolongation was demonstrated by Lazzerini et al. in a cohort of 101 patients with chronic inflammatory arthritis, and this relationship was further linked to C-reactive protein (CRP), circulating IL-6 levels, and other inflammatory cytokines in RA [31,32,33]. The mechanisms by which circulating inflammatory cytokines prolong the QTc remain uncertain, as these mediators may exert direct effects on the myocardium or act indirectly by enhancing the central sympathetic drive to the heart [34].

The FIB-4 index was not used as a diagnostic surrogate for non-alcoholic fatty liver disease (NAFLD) in this study but rather as a non-invasive marker integrating age and routine biochemical parameters that reflect subclinical hepatic fibrosis and systemic inflammatory–metabolic burden. Although overt NAFLD was not formally assessed, elevated FIB-4 values may capture chronic low-grade inflammation, oxidative stress, and endothelial dysfunction—processes that are also implicated in ventricular electrical remodeling and arrhythmic risk. In this context, the observed association between FIB-4 and QTc likely reflects shared pathophysiological pathways rather than a direct liver-specific mechanism. This concept is consistent with emerging evidence describing a bidirectional liver–heart axis mediated by inflammatory cytokines, metabolic dysregulation, and fibrotic signaling pathways that may influence both hepatic and cardiac structure and function [35,36,37,38,39]. Although the association between the FIB-4 index and QTc was statistically significant, its magnitude was moderate (r = 0.475), corresponding to an explained variance of approximately 22%. This indicates that FIB-4 accounts for only a limited proportion of QTc variability, which is consistent with the multifactorial nature of ventricular repolarization. QTc duration is influenced by multiple interacting factors, including autonomic tone, myocardial structure, electrolyte balance, renal function, medications, and systemic inflammatory activity. Therefore, FIB-4 should be interpreted not as a dominant determinant of QTc but as an independent risk marker reflecting one component of a broader inflammatory–fibrotic milieu contributing to electrical remodeling in patients with ankylosing spondylitis.

The FIB-4 index was selected not as a marker of acute inflammation but as a surrogate of chronic cumulative inflammatory–fibrotic burden. Classical inflammatory biomarkers such as C-reactive protein and circulating interleukins primarily reflect short-term inflammatory activity and fluctuate with disease activity and treatment, whereas FIB-4 integrates age, aminotransferase levels, and platelet count—variables influenced by long-standing immune activation, low-grade inflammation, and tissue remodeling. In our cohort, C-reactive protein did not show a significant association with QTc, whereas FIB-4 did, suggesting that chronic inflammatory–fibrotic burden rather than acute inflammatory activity may be more closely linked to ventricular electrical remodeling. Therefore, FIB-4 was used as a complementary marker representing a different biological dimension than classical inflammatory cytokines.

In the univariable analyses, the significant negative association observed between eGFR and QTc was consistent with previous studies demonstrating the influence of renal function on ventricular repolarization [40]. However, this relationship did not remain independent in the multivariate model. Similarly, in agreement with several reports in the literature, we identified a negative correlation between hemoglobin levels and QTc. Anemia is associated with elevated levels of reactive oxygen species, oxidative stress, and low-grade chronic inflammation, and multiple studies have suggested that these factors, together with ceramide-related pathways, may contribute to QT interval prolongation [41,42,43].

Recent evidence has further highlighted the multifactorial determinants of QTc prolongation. A new study has shown that proteomic markers associated with myocardial fibrosis and angiogenesis are associated with QTc prolongation, suggesting a potential mechanistic bridge between structural cardiac remodeling and chronic inflammation [44]. Similarly, the clinical evaluation of drug-induced QT prolongation emphasizes that QTc risk is not solely dependent on inflammation but involves a combination of electrophysiological susceptibility, metabolic factors, and external triggers, reinforcing the need for multifactorial assessment in patients with AS and comorbid liver disease [45]. Furthermore, exercise-induced QT prolongation highlights that certain QTc changes may be reversible and distinguishable from congenital Long QT Syndrome [46]. This underscores the dynamic nature of QTc regulation and suggests that autonomic and activity-related factors may modulate QTc in AS patients, potentially interacting with inflammatory and metabolic pathways. Taken together, these new findings strengthen the notion that QTc prolongation in AS patients with elevated FIB-4 may reflect a complex interplay of chronic inflammation, hepatic fibrosis, autonomic modulation, and structural cardiac remodeling. These insights provide a rationale for closer cardiovascular monitoring and individualized risk stratification in this population.

The findings of prolonged QTc in AS patients with elevated FIB-4 also raise considerations regarding primary and secondary prevention of life-threatening ventricular arrhythmias. Although no guidelines specifically address ICD implantation in AS, extrapolation from data in inflammatory and cardiomyopathy populations suggests that patients with markedly prolonged QTc, documented ventricular arrhythmias, or additional high-risk features (e.g., significant myocardial fibrosis, reduced ejection fraction, or prior syncope) may benefit from an ICD for sudden cardiac death prevention. Regarding device selection, transvenous ICDs remain the standard choice when pacing or anti-tachycardia therapies are needed, especially in patients with conduction disturbances or bradyarrhythmias. However, subcutaneous ICDs (S-ICDs) are increasingly considered in patients with preserved pacing requirements but high arrhythmic risk, as they reduce lead-related complications, particularly in younger patients or those with systemic inflammatory disease who may have venous or vascular issues [47].

Emerging evidence indicates that in patients with systemic inflammatory conditions, including AS, careful patient selection is crucial: S-ICDs may minimize infection and lead failure risk, whereas transvenous systems provide more versatile therapy for bradyarrhythmia and anti-tachycardia pacing. Guideline recommendations from major societies support S-ICD use in patients with an ICD indication but without pacing requirements [48,49]. Ultimately, the choice of device should integrate arrhythmic risk stratification, structural cardiac findings, and comorbid conditions such as NAFLD or renal dysfunction, which may further modulate QTc and arrhythmic susceptibility.

## 5. Limitations

This study had several limitations. The sample size was relatively small, and the analysis was conducted at a single center, which may limit the generalizability of the findings. Owing to its observational cross-sectional design, exposure and outcome were assessed at a single time point; therefore, no temporal or causal inferences could be made. The absence of a control group restricted our ability to determine whether the associations observed were specific to AS. Residual confounding is possible, as disease duration, cumulative medication exposure, and fluctuations in inflammatory activity could not be fully characterized in this study. In addition, hepatic steatosis, or metabolic dysfunction–associated fatty liver disease, was not directly assessed using imaging or dedicated diagnostic criteria; therefore, the FIB-4 index should be interpreted as a systemic fibrosis/inflammation marker rather than as evidence of underlying liver disease.

Subclinical hepatic dysfunction and other unmeasured factors may have also influenced the results. QTc was calculated using Bazett’s formula, and alternative correction methods were not considered. Because of the cross-sectional design, the observed associations cannot establish causality or mechanistic pathways and should be interpreted as hypothesis-generating rather than explanatory. Future studies combining acute inflammatory markers (e.g., CRP, interleukins), imaging of myocardial and hepatic fibrosis, and longitudinal ECG follow-up will be necessary to disentangle causal mechanisms.

## 6. Conclusions

This study demonstrated that the FIB-4 index is independently associated with the QTc in patients with AS. These findings suggest that FIB-4, a simple and noninvasive parameter reflecting systemic fibrotic–inflammatory burden, may provide additional information for cardiovascular risk stratification in this population. Given that QTc prolongation increases the risk of ventricular arrhythmias and SCD, patients with AS who exhibit elevated FIB-4 values may warrant closer cardiac monitoring and the consideration of preventive strategies.

## Figures and Tables

**Table 1:** Demographic and clinical characteristics of the study population (*n* = 82).

Variable	Value
Age, years	42.4 ± 11.7
Male sex, *n* (%)	47 (57.3)
Diabetes mellitus, *n* (%)	13 (15.9)
Hypertension, *n* (%)	18 (22.0)
Hyperlipidemia, *n* (%)	43 (52.4)
Coronary artery disease, *n* (%)	4 (4.9)
Current smoking, *n* (%)	31 (38.3)
BASDAI score	4.1 ± 2.3
HLA-B27 positivity, *n* (%)	46 (56.8)
FIB-4 index	0.75 ± 0.32
β-blocker use, *n* (%)	6 (7.3)
Calcium-channel blocker use, *n* (%)	10 (12.2)
ACE inhibitor use, *n* (%)	8 (9.8)
ARB use, *n* (%)	8 (9.8)
Statin use, *n* (%)	5 (6.1)
Oral antidiabetic use, *n* (%)	3 (3.7)
eGFR, mL/min/1.73 m^2^	105.6 ± 15.9
Serum creatinine, mg/dL	0.78 ± 0.17
Glucose, mg/dL	98.9 ± 17.9

Values are presented as mean ± standard deviation or number (percentage), as appropriate. Abbreviations: BASDAI, Bath Ankylosing Spondylitis Disease Activity Index; HLA-B27, human leukocyte antigen B27; FIB-4, Fibrosis-4 Index; ACE, angiotensin-converting enzyme; ARB, angiotensin receptor blocker; eGFR, estimated glomerular filtration rate.

**Table 2:** Correlation analysis between QTc interval and clinical/laboratory variables (*n* = 82).

Variable	r	*p*-Value
Age	0.498	<0.001 *
Sex	−0.284	0.011 *
HLA-B27	NS	NS
BASDAI score	−0.217	0.053
FIB-4 index	0.475	<0.001 *
Body mass index	0.254	0.025 *
Diabetes mellitus	NS	NS
Hypertension	0.342	0.002 *
Hyperlipidemia	0.277	0.013 *
Coronary artery disease	NS	NS
Current smoking	NS	NS
β-blocker use	0.246	0.028 *
Calcium-channel blocker use	0.399	<0.001 *
ACE inhibitor use	0.264	0.018 *
ARB use	0.204	0.070
Statin use	0.297	0.007 *
White blood cell count	NS	NS
Neutrophils	NS	NS
Lymphocytes	NS	NS
Hemoglobin	−0.225	0.045 *
Glucose	NS	NS
Serum creatinine	NS	NS
eGFR	−0.368	<0.001 *
C-reactive protein	NS	NS
Pulse wave velocity	NS	NS
Flow-mediated dilation	NS	NS
Right CIMT	NS	NS
Left CIMT	NS	NS

NS indicates a non-significant correlation. * *p* < 0.05 was considered statistically significant. Abbreviations: BASDAI, Bath Ankylosing Spondylitis Disease Activity Index; FIB-4, Fibrosis-4 Index; ACE, angiotensin-converting enzyme; ARB, angiotensin receptor blocker; eGFR, estimated glomerular filtration rate; CIMT, carotid intima–media thickness.

**Table 3:** Multivariable linear regression analysis for predictors of QTc interval.

Variable	Unstandardized β (B)	Standardized β	t	95% CI for B	*p*-Value
Sex	−14.577	−0.306	−3.308	−23.352 to −5.802	0.001
FIB-4 index	27.822	0.379	3.470	11.854 to 43.790	<0.001

Model includes variables that remained significant after univariable analysis. CI, confidence interval; FIB-4, Fibrosis-4 Index. *p* < 0.05 was considered statistically significant.

## Data Availability

The data presented in this study are available upon reasonable request from the corresponding author.
